# Development of an Ammonium Sulfate DNA Extraction Method for Obtaining Amplifiable DNA in a Small Number of Cells and Its Application to Clinical Specimens

**DOI:** 10.1155/2013/546727

**Published:** 2013-04-17

**Authors:** Seo Young Oh, Wook Youn Kim, Tae Sook Hwang, Hye Seung Han, So Dug Lim, Wan Seop Kim

**Affiliations:** ^1^Department of Animal Biotechnology, College of Animal Bioscience and Technology, Konkuk University, 1 Hwayang-dong, Gwangjin-gu, Seoul 143-701, Republic of Korea; ^2^Department of Pathology, Konkuk University School of Medicine, 1 Hwayang-dong, Gwangjin-gu, Seoul 143-701, Republic of Korea

## Abstract

DNA extraction from microdissected cells has become essential for handling clinical specimens with advances in molecular pathology. Conventional methods have limitations for extracting amplifiable DNA from specimens containing a small number of cells. We developed an ammonium sulfate DNA extraction method (A) and compared it with two other methods (B and C). DNA quality and quantity, **β**-globin amplification, and detectability of two cancer associated gene mutations were evaluated. Method A showed the best DNA yield, particularly when the cell number was very low. Amplification of the **β**-globin gene using DNA from the SNU 790 cell line and papillary thyroid carcinoma (PTC) cells extracted with Method A demonstrated the strongest band. *BRAF* ^*V600E*^ mutation analysis using ethanol-fixed PTC cells from a patient demonstrated both a “T” peak increase and an adjacent “A” peak decrease when 25 and 50 cells were extracted, whereas mutant peaks were too low to be analyzed using the other two methods. *EGFR* mutation analysis using formalin-fixed paraffin-embedded lung cancer tissues demonstrated a mutant peak with Method A, whereas the mutant peak was undetectable with Methods B or C. Method A yielded the best DNA quantity and quality with outstanding efficiency, particularly when paucicellular specimens were used.

## 1. Introduction

Molecular diagnosis combines molecular biology techniques with knowledge of molecular mechanisms of the disease and helps to diagnose causes of illness and to make a therapeutic decision. It usually uses diagnostic assays based on direct extraction of DNA or RNA from tissue sections or cytological preparations [1].

In clinical practice, a molecular diagnosis often requires isolation of genomic DNA from paucicellular clinical specimens such as fine needle aspirates, nipple fluid aspirates, sputum, bronchial wash fluid, buccal swabs, cervical smears, or urine [2–7]. If high-quality amplifiable DNA can be extracted from these specimens, various genetic tests including LOH analysis, gene copy number determination, genotyping, mutation analysis, or promoter methylation studies can be conducted for a molecular diagnosis [8–13]. As microdissected cells from clinical specimens have become important target material for molecular pathology, efficient genomic DNA extraction from a small number of microdissected cells has become a critical step for amplifying DNA [14]. Isolating DNA from a very small specimen using traditional methods is difficult and inefficient primarily due to DNA loss during filtration and precipitation. This often results in amplification failure when the quantity of starting material is limited. Several commercial DNA extraction kits have become available and have been promoted as alternatives to organic extraction methods to prepare DNA for amplification. They include adsorption of DNA on a silica membrane (Pinpoint Slide DNA Isolation System), an ion exchange resin (TaKaRa DEXPAT), and a salting-out procedure (Wizard Genomic DNA Purification Kit). As most extraction methods have a relatively low efficiency rate, van Oorschot et al. suggested to optimize methodologies specifically for trace samples so that one has the opportunity to (a) extract most of, if not all, the available DNA; (b) remove all amplification inhibiting elements without the loss of DNA; (c) utilize all of the extracted DNA for amplification; and (d) add the amplification reagents to the vessel containing the DNA rather than having to transfer the DNA to a separate vessel containing the amplification reagents (thus avoiding a further loss that could be encountered in the transfer step) [15].

The Pinpoint Slide DNA Isolation System was developed to easily isolate genomic DNA in any targeted microscopic tissue on a slide and is being used in laboratories handling trace samples particularly in forensic pathology [16, 17]. TaKaRa DEXPAT was designed to enable quick-step extraction of polymerase-chain-reaction- (PCR-) ready DNA from formalin-fixed paraffin-embedded (FFPE) tissue and small specimens and is currently used in many molecular diagnostic laboratories [18]. However, these kits have some limitations, as loss of DNA has been attributed to multiple steps (Pinpoint slide DNA isolation System) or protein contamination due to the absence of a digestion step (TaKaRa DEXPAT), particularly when samples are very small [16–18]. Therefore, obtaining amplifiable DNA using these commercial kits is often unsatisfactory due to the frequent handling of small specimens containing only a few clusters of target cancer cells. In fact, accurate evaluation data of these kits for isolating DNA from a small specimen has not been provided. Important factors to consider when evaluating extraction procedures include DNA yield and purity, suitability of the DNA for amplification, the number of transfer steps, time, and cost of the method [19].

To overcome these difficulties in clinical practice, we developed an ammonium sulfate DNA extraction method. In this study, we compared the quantity and quality of DNA extracted with this ammonium sulfate  DNA  extraction method with those of DNA extracted using two commercially available kits and a spectrophotometer readout for **β*-globin* amplification, *BRAF *(v-raf murine sarcoma viral oncogene homolog B1)^*V600E*^, and epithelial growth factor receptor (*EGFR*) mutation analysis.

## 2. Materials and Methods

### 2.1. Specimen Preparation

#### 2.1.1. Preparation of Human Papillary Thyroid Carcinoma (PTC) Cell Line

The SNU 790 human PTC cell line obtained from the Korean Cell Line Bank (Cancer Research Institute, Seoul National University, Seoul, Republic of Korea) was used. The cells were maintained in RPMI-1640 medium (Gibco, Grand Island, NY, USA) containing heat-inactivated 10% fetal bovine serum (Gibco). The cultures were maintained in humidified incubators at 37°C in an atmosphere of 5% CO_2_ and 95% air. The cells were collected by centrifugation at 1,500 rpm for 5 minutes. The cell pellet was resuspended in 10 mL of fresh medium and counted using a hemocytometer. Tumor cells were diluted with fresh medium to cell concentrations of 50, 100, 200, 500, and 1000 cells/mL. After centrifugation at 1500 rpm for 10 minutes, the supernatant was discarded and 50 *µ*L of each extraction buffer was added. The whole procedure was performed in triplicate after the tumor cells were harvested from the culture media.

#### 2.1.2. Preparation of Nonparaffinized Cells on Archival Smear Slides

PTC cells from archival smear slides aspirated from the same patient in the Department of Pathology files of Konkuk University Medical Center were used. Study approval was obtained from the Institutional Review Board (KUH 1210027). The tumor cells were aspirated from the thyroid gland with a 26-gauge needle under ultrasound guidance and were smeared on glass slides and fixed with 95% ethanol. The fixed cells were stained using the Papanicolau procedure. After the target cells were marked by the cytopathologist, the cover slides were removed in xylene and air-dried. The slides were rinsed in 2% glycerol in TE buffer (10 Mm Tris-HCl pH 8.5, 1 mM EDTA, pH 8.0) for 2 minutes. The target cells were dissected with a 26-gauge needle under 100× magnification. Approximately, 25, 50, 100, 200, 500, and 1000 cells were dissected using a square micrometer in the microscope. A needle tip was carefully submerged in a tube containing 50 *µ*L of each extraction buffer to collect the cells. For all cell number groups, the cells were collected three times for nucleic acid and *BRAF* ^*V*600*E*^ mutation analyses and ten times to evaluate **β*-globin* gene amplification.

#### 2.1.3. Preparation of FFPE Tissue

No preparation step was required for Method A, whereas deparaffinization was required for Methods B and C. For tissue deparaffinization, 200 *µ*L xylene was added to whole, half, and quarter portions of one tissue section (2 mm circle of 4 *µ*m thickness) and agitated for 5 minutes, then centrifuged at 12,000 rpm for 10 minutes. The supernatant was removed and fresh xylene was added, and this step was repeated three times followed by washing with 100% ethanol for 10 minutes and centrifuging at 12,000 rpm for 10 minutes. Then, the tissue pellet was air-dried.

Fifty microliters of DNA extraction buffer solution from each protocol was added to the deparaffinized tissue in a 1.5 mL microcentrifuge tube followed by the DNA extraction step. DNA extraction without the deparaffinization step was also performed. The tissues were collected three times for *EGFR *mutation analysis.

### 2.2. DNA Extraction

#### 2.2.1. Method A

Briefly, 50 *μ*L of ammonium sulfate DNA extraction buffer solution (16 mM (NH_4_)_2_SO_4_, 50 mM Tris-HCl pH 8.5, 1 mM EDTA pH 8.0, and 0.5% Tween-20) was added to the dissected cells or tissues in a 0.2 mL PCR tube, and Proteinase K (Takara Bio. Inc., Shiga, Japan) was added to a final concentration of 200 *µ*g/mL and then digested at 56°C for 1 hour. Following the incubation, the PCR tube was heated to 100°C for 20 minutes in a PTC-220 Thermocycler (Bio-Rad, Hercules, CA, USA) with 10% Chelex-100 (Bio-Rad) after gentle shaking and centrifuged at 12,000 rpm for 10 minutes to elute the DNA.

#### 2.2.2. Method B

Dissected cells and tissues were treated according to the manufacturer's protocol for the Pinpoint Slide DNA Isolation System (Zymo Research, Irvine, CA, USA). Briefly, 50 *μ*L of DNA extraction buffer was added to the 1.5 mL tube containing dissected cells or tissues and digested at 56°C for 4 hours after 5 *μ*L of Proteinase K was added. After adding 100 *μ*L of Pinpoint Binding Buffer, transfer to a Zymo-Spin1 Column and span the Column at 12000 rpm for 10 seconds. After washing 2 times by adding PP Wash Buffer to the Zymo-Spin1 Column and spining at 12000 rpm for 10 seconds, transfer the Column into a new 1.5 mL tube. Then, add the TE Buffer and span at 12000 rpm for 10 seconds to elute the DNA.

#### 2.2.3. Method C

Dissected cells or tissues were heated to 100°C for 10 minutes with 50 *μ*L of resin-based TaKaRa DEXPAT solution (Takara Bio. Inc., Shiga, Japan). The tube was centrifuged at 12,000 rpm for 10 minutes to elute the DNA.

### 2.3. Evaluation of Nucleic Acids

Each extract was brought to a final volume of 50 *µ*L and the A_260_ and A_280_ were measured after diluting 2 *µ*L of the extract in 498 *µ*L of water using a DU-500 spectrophotometer (Beckman Coulter, Brea, CA, USA). DNA purity was determined by calculating the absorbance A_260_/A_280_ ratio. Pure DNA has a ratio of 1.8 ± 0.2 [20, 21]. The yield and purity of DNA from the SNU 790 cell line and PTC cells on the archival smear slides were measured from three specimens in each cell number group, and the mean value was calculated.

### 2.4. PCR Amplification of the **β*-Globin* Gene

PCR amplification was performed using the SNU 790 cell line and PTC cells on archival smeared slides. The forward primer PC-3, 5′-ACACAACTGTGTTCACTAGC-3′ and reverse primer PC-4, 5′-CAACTTCATCCACGT TCACC-3′ were used to amplify a 110 bp DNA fragment. Briefly, 2 *μ*L of DNA was added to a total 20 *μ*L PCR solution mixture containing 0.2 mM of each dNTP, 1.5 mM MgCl_2_, 1 × PCR buffer, 1.5 U of Immolase DNA Taq. Polymerase (Bioline, London, UK), and 20 pmol of each primer. PCR was performed with an initial denaturation for 10 minutes at 95°C followed by five cycles (1 minute at 95°C, 1 minute at 55°C, 1 minute at 72°C), 35 cycles (1 minute at 95°C, 1 minute at 55°C, 1 minute at 72°C), and a 10-minute incubation at 72°C. The PCR products were analyzed by agarose gel electrophoresis to confirm successful amplification. **β*-Globin* gene amplification PCR was performed from ten specimens in each cell number group.

### 2.5. Validation of **β*-Globin* Gene Amplification

Approximately 25, 50, 100, 200, 500, and 1000 cells were scraped, and DNA was extracted from 95% alcohol-fixed (Merck KGaA, Darmstadt, Germany) PTC cells on archival smear slides using the three different methods. The amplified template was analyzed using the MultiNA Microchip Electrophoresis System DNA-500 kit (Shimadzu Corp., Tokyo, Japan). **β*-Globin* gene amplification was performed as an internal control.

### 2.6. *BRAF* ^*V*600*E*^ Mutation Analysis Using Pyrosequencing

The SNU 790 cell line and PTC cells on archival smear slides were used for analysis. The primer sequences for amplification of the *BRAF* ^*V*600*E*^ mutation site on exon 15 were as follows: 5′-biotin-CTTCATAATGCTTGCTCTGATAGG-3′ (F) and 5′-GGCCAAAAATTTAATCAGTGGAA-3′ (R). Five microliters of DNA was added to a total 50 *μ*L PCR solution mixture containing 0.2 mM of each dNTP, 1.5 mM MgCl_2_, 1 × PCR buffer, 1.5 U of Immolase DNA Taq polymerase, and 20 pmol of each primer. PCR was carried out with an initial denaturation for 5 minutes at 95°C followed by 40 cycles (30 seconds at 95°C, 30 seconds at 55°C, and 30 seconds at 72°C) and a 10-minute incubation at 72°C. The PCR products were analyzed by agarose gel electrophoresis to confirm successful amplification.

The biotinylated products were then immobilized to streptavidin-coated beads using the solution from a commercial PSQTM96 Sample Preparation kit. Three microliters of beads were diluted in binding buffer with 10 *μ*L of biotinylated PCR products and incubated for 10 minutes at room temperature. The beads was then transferred to a filter probe, and liquid was removed by vacuum filtration. The DNA in the denaturation solution was separated, templates were washed with washing buffer, transferred to a PSQ 96 SNP plate, and annealed with a sequencing primer (5′-CCACTCCATCGAGATTT-3′) in buffer at room temperature. Finally, the specimens were analyzed using a PyroMark ID System with a SNP reagent kit for sequencing [22, 23]. The *BRAF* ^*V600E*^ mutation analysis was performed with three specimens from each cell number group.

### 2.7. *EGFR *Mutation Analysis Using Pyrosequencing

FFPE human lung cancer tissues were used for this analysis. The primer sequences for amplification of the *EGFR* mutation site on exon 19 and sequencing were 5′-GCATGTGGCACCATCTCA-3′(F), 5′-biotin-AAAAGGTGGGCCTGAGGTT-3′(R), and 5′-ATTCCCGTCGCTATC-3′, respectively. Whole procedure was performed as previously described for the BRAF mutation analysis except for the annealing condition (30 seconds at 60°C).

### 2.8. Statistical Analysis

We used analysis of variance to compare the means of DNA yields from the three different DNA extraction methods. Differences in **β*-globin* amplification among the DNA extraction methods were analyzed by the two-tailed Fisher's exact test and verified by Student's *t*-test. A *P*  value <0.05 was considered significant.

## 3. Results

### 3.1. Nucleic Acid Evaluation

Table 1 shows the mean total amount, concentration, and purity of DNA using the three different techniques in various numbers of cells from the SNU 790 cell line and PTC cells on archival smear slides.

After analyzing the DNA from the SNU 790 cell line, the mean concentration (mean total amount) of DNA ranged from 0.124 to 1.125 ng/*µ*L (6.180 to 57.653 ng) for Method A, 0.067 to 0.451 ng/*µ*L (3.367 to 22.527 ng) for Method B, and 0.066 to 0.761 ng/*µ*L (3.320 to 38.033 ng) for Method C. A_260_ values ranged from 0.0025 to 0.0231 for Method A, 0.0013 to 0.0090 for Method B, and 0.0013 to 0.0152 for Method C. The purity (A_260_/A_280_) of DNA ranged from 1.5 to 1.8 for Method A, 1.6 to 1.8 for Method B, and 1.4 to 1.6 for Method C. Method A showed a better yield than that of the other two methods for all specimens (Table 1).

After analyzing the PTC cells on archival smear slides, the mean concentration (mean total amount) of DNA ranged from 0.013 to 0.406 ng/*µ*L (0.652 to 20.280 ng) for Method A, 0.001 to 0.242 ng/*µ*L (0.070 to 12.115 ng) for Method B, and 0.004 to 0.307 ng/*µ*L (0.190 to 15.347 ng) for Method C. A_260_ values ranged from 0.0003 to 0.0081 for Method A, 0.0000 to 0.0048 for Method B, and 0.0001 to 0.0061 for Method C. The purity (A_260_/A_280_) of DNA ranged from 1.5 to 1.8 for Method A, 1.2 to 1.8 for Method B, and 1.3 to 1.7 for Method C. Method A provided a DNA yield up to nine times greater when the cell number was very small (25–50 cells) (Table 2).

### 3.2. **β*-Globin* Gene Amplification Status Using DNA from Samples with Various Numbers of Cells

**Figure 1:**
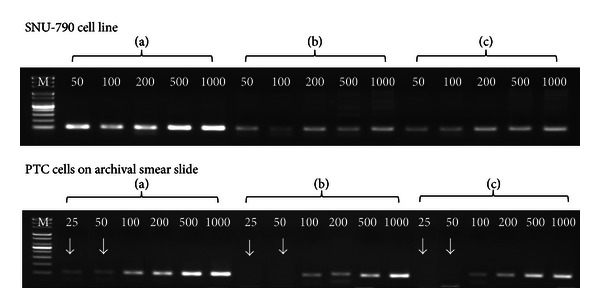
Comparison of **β*-globin *gene amplification using DNA extracted from various numbers of SNU 790 cells and PTC cells on archival smear slides. We compared PCR products amplified from DNA extracted using three different methods to determine the efficacy of DNA extraction. This assay used **β*-globin* primers that amplify a 110 bp fragment of genomic DNA. (a) Ammonium sulfate DNA extraction method; (b) Pinpoint Slide DNA Isolation System; (c) TaKaRa DEXPAT; PTC: papillary thyroid carcinoma.

**β*-Globin* gene amplification status was analyzed to compare the quality of the methods for extracting DNA from various numbers of SNU 790 cells and PTC cells on archival smear slides. Amplification of the **β*-globin* gene using DNA from the SNU 790 cell line extracted with Method A demonstrated the strongest band, whereas Methods B and C produced weaker and irregular bands for all specimens. Method A demonstrated an amplified product when 25 and 50 PTC cells from the archival smear slide were used, whereas at least 100–200 cells were required for amplification using Methods B and C (Figure 1).

**Figure 2:**
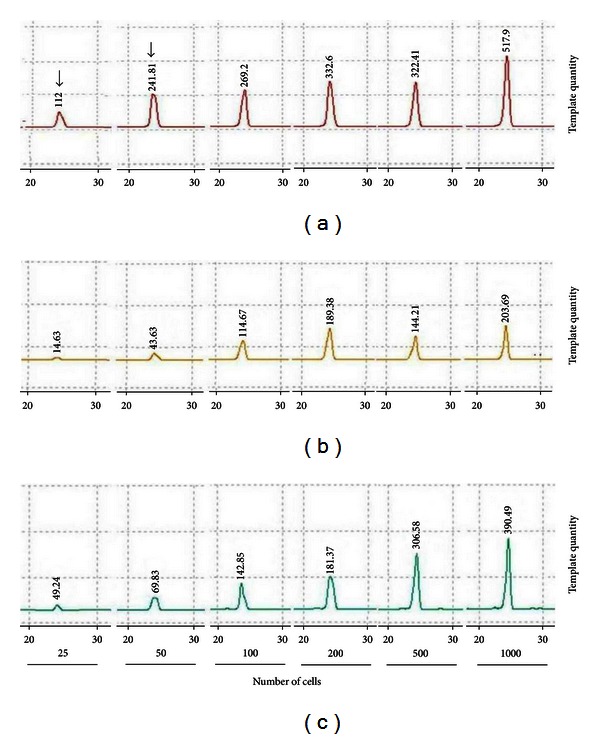
Comparison of template quantity during **β*-globin *gene amplification using various numbers of PTC cells on archival smear slides. (a) Ammonium sulfate DNA extraction method; (b) Pinpoint Slide DNA Isolation System; (c) TaKaRa DEXPAT; PTC: papillary thyroid carcinoma.

To verify the efficiency of amplification extracted with the three methods, we compared the success rate of **β*-globin* gene amplification using various numbers of target cells.  Table 3 shows the successful **β*-globin* gene amplification status using DNA from PTC cells on archival smear slides. Method A produced an amplifiable **β*-globin* gene more frequently than that of the other methods when starting cell counts were <200  (*P* = 0.002). The amplified **β*-globin* gene was similar for all methods when starting cell number was ≥500  (*P* = 0.002). When 25 and 50 cells were extracted using Method A, successful **β*-globin* gene amplification was significantly higher than that with Methods B or C (*P* = 0.011). To verify the superiority of Method A, we quantified the template amount using the MultiNA Microchip Electrophoresis System DNA-500 Kit (Figure 2). Method A produced higher **β*-globin* template quantity than that of the other methods for all numbers of cells.

### 3.3. Determining the Efficiency of the Mutation Analysis in Nonparaffinized Cells

**Figure 3:**
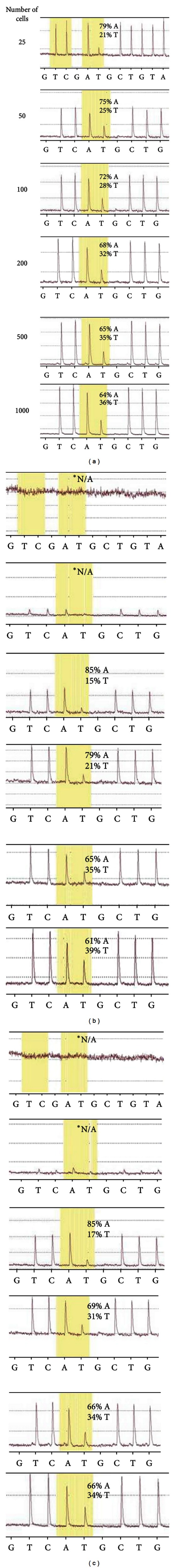
Comparison of the efficiency of DNA extraction by examining the *BRAF* ^*V*600*E*^ mutation detectability in various numbers of PTC cells on archival smear slides. (a) Ammonium sulfate DNA extraction method; (b) Pinpoint Slide DNA Isolation System; (c) TaKaRa DEXPAT; PTC: papillary thyroid carcinoma; *N/A: not applicable.

We analyzed the *BRAF* ^*V*600*E*^ mutation in different numbers of target cells to confirm the efficiency of Method A in cytology specimens. Data using various numbers of PTC cells on archival smear slides are shown in Figure 3. The intensity of the “T” peak signal height ranged from 21 to 36% with Method A, 0 to 39% with Method B, and 0 to 34% with Method C. When 25 and 50 cells were extracted, Method A detected both the increase in the “T” peak (21 and 25%, resp.) and the decrease in the adjacent “A” peak (79 and 75%, resp.), whereas sequence peaks were too low to be analyzed by the other two methods. When 100 cells were used, Method A showed a significant increase in the “T” peak and a decrease in the “A” peak, and the “T” peak signal began to appear in Methods B and C. When 200 cells were used, Methods B and C showed the “T” peak signal, although the signal was lower than that of Method A. No significant difference was observed among the three methods when 500 cells were extracted.

In contrast, all three methods were able to identify mutant peaks in the *BRAF* ^*V600E*^ analysis using the SNU 790 cell line, although the mutant peak was very low in Methods B and C when 50 cells were used. Taken together, Method A provided superior efficiency when sequencing a small number of cells.

### 3.4. Determining the Efficiency of the Mutation Analysis in FFPE Tissue

**Figure 4:**
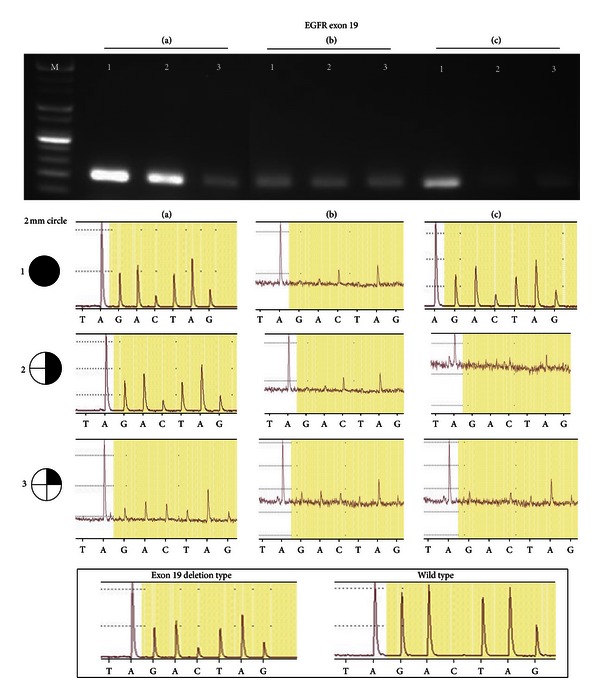
Comparison of the efficiency of DNA extraction by examining the *EGFR *mutation in different amounts of FFPE tissue. (a) Ammonium sulfate DNA extraction method; (b) Pinpoint Slide DNA Isolation System; (c) TaKaRa DEXPAT; PTC: papillary thyroid carcinoma. 1: whole tissue; 2: 1/2 of one section; 3: 1/4 of one section (2 mm circle with a 4 *µ*m thickness).

To confirm the efficiency of Method A in FFPE tissue specimens, we analyzed the *EGFR *mutation in different amounts of FFPE human lung cancer tissue using the PyroMark ID System (Figure 4). Amplification of the *EGFR* gene using DNA extracted by Method A demonstrated a mutant peak signal with detectable height and a stable baseline in all specimens with a strong band shown in whole and half tissue sections but a relatively weak band for the quarter tissue section. Method B, with a deparaffinization step, showed a weak band and an undetectable mutant peak for all specimens. Method C, with a deparaffinization step, showed the mutant peak signal only when the whole tissue section was used. No band was detected when Methods B and C were used to amplify the *EGFR* gene (data not shown).

## 4. Discussion

Molecular diagnosis provides important information about the molecular characteristics of a disease, which is critical for diagnostic and therapeutic decision-making. In this respect, obtaining high-quality amplifiable DNA from cytological specimens or small biopsy specimens containing, only a few target cells is very important to prevent repeated invasive procedures in clinical practice.

In this study, we compared Method A with two different commercial DNA extraction methods using cell lines, microdissected cells smeared on glass slides, and FFPE tissues. DNA extraction from these microdissected cells was quite challenging.

DNA quantity and quality were estimated by DNA concentration and DNA purity. The quantity of nucleic acids in a solution is often estimated based on the absorbance of light at a wavelength of 260 nm. An A_260_ value of 1.0 correlates roughly with double-stranded DNA content of 50 *μ*g/mL. The A_280_ value is traditionally taken as a measure of protein content in a solution (though nucleic acids absorb a considerable amount of light at 280 nm), and the A_260_/A_280_ ratio is a measure of nucleic acid extract purity. An A_260_/A_280_ ratio of 1.8 ± 0.2 is generally considered relatively free of protein contamination [24, 25]. Method A yielded the highest DNA concentration (Table 1) and the best amplified **β*-globin* gene products when all cell number quantities were tested, particularly for the smallest cell (25 cells) number group (*P* = 0.011) (Table 3 and Figure 1). DNA purity obtained by Method A was in the 1.5 to 1.8 range and demonstrated little protein contamination. Method A was superior to the other two methods when applied to alcohol-fixed archival cytology specimens as determined by the peak on the MultiNA microchip obtained by electrophoresing **β*-globin* template quantity (Figure 2).

The pyrosequencing assay was designed to begin sequence analysis immediately at the mutation site. A sequential nucleotide dispensation protocol was used that reflects the expected order of nucleotide incorporation and the potential base changes. Peak signal heights are proportional to the number of nucleotides that are incorporated with each dispensation [26]. When the *BRAF* ^*V*600*E*^ mutation analysis was used to determine the efficiency of DNA extraction, no significant difference in the sequence change was observed among all three methods when the SNU 790 cell line was used. However, Figure 3 shows significant differences for detecting the mutation when PTC cells on archival smear slides were used. Notably, both the increase in “T” peak signal height and decrease in the adjacent “A” peak signal height were present when 25 and 50 cells were used in Method A. This was in contrast to the two other methods where sequence peaks were too low to be observed (Figure 3). These results suggest that our ammonium sulfate DNA extraction method provided superior quality and yield in paucicellular clinical specimens.

The increased yields of Method A may be attributed to the extraction buffer components, the appropriate concentration of Proteinase K (200 *µ*g/mL), and optimum temperature. The alkaline (pH 8–11) of the extraction buffer containing ammonium sulfate aided protein precipitation due to hydrophobic interactions and ultimately provided good DNA quantity and quality. A precise amount of Proteinase K in the extraction buffer and optimum reaction temperature promote the inactivation of nucleases and DNA release from target cells [27–29]. High-temperature heating and an alkaline solution have been used to improve nucleic acid extraction from archival smeared cells and FFPE tissues and provide the best results as indicated by both the quantity and quality of DNA yield [30]. These conditions help to denature and hydrolyze proteins, resulting in rupture of cellular and nuclear membranes and breakage of cross-links introduced by fixation [30, 31].

Because clinical specimens often have a limited number of cells, a reproducible and cost-effective DNA extraction method is absolutely needed. Method A demonstrated the possibility of successful molecular genetic testing even with 25 target cells. This minimum requirement of 25 cells is overwhelmingly advantageous, as it provides the ability to apply various molecular techniques to cytological specimens. If cytological specimens such as bronchial wash specimens could replace lung biopsy specimens when analyzing *EGFR* mutation status in patients with inoperable lung cancer, it would be a powerful option, as repeated lung biopsy could be avoided in patients with poor clinical condition. In addition to the cytological specimens, histological specimens could also benefit by applying Method A. Even small biopsy specimens containing only a few clusters of target cells could be utilized for molecular analysis. In practice, small biopsy specimens are often not available for additional genetic testing. In that case, genetic tests can still be performed using previously stained slides.

In addition to these benefits, Method A also has practical advantages of being faster, easier, and simpler than Method B (required 1 hour of deparaffinization, a 4-hour digestion, and complicated DNA purification steps). The procedures of Method C are as simple and easy as Method A. However, this method does not have Proteinase K digestion step which is critical for protein lysis. This method also required a deparaffinization step, which is not necessary in Method A (Figure 4). These benefits can save significant time and effort when conducting DNA extractions in hospital-based molecular pathology laboratories.

In conclusion, Method A yielded the highest quantity and quality of DNA with outstanding efficiency particularly when paucicellular specimens were used. In addition, this extraction method provides an opportunity to apply molecular techniques to small FFPE tissue and archival cytology specimens.

## Figures and Tables

**Table 1:** Comparison of DNA yield and purity extracted using three different methods from various numbers of SNU 790 cell line.

No. of cells	Extraction method	^¶^DNA concentration (ng/*μ*L)	^¶^Total DNA yield (ng)	^¶^Purity A_260_/A_280_	*P* value*
50 (*n* = 3)	A	0.124 ± 0.005	6.180 ± 0.38	1.7 ± 0.1	*P* = 0.001
	B	0.067 ± 0.004	3.367 ± 0.39	1.2 ± 0.5	
	C	0.066 ± 0.006	3.320 ± 0.39	1.3 ± 0.1	

100 (*n* = 3)	A	0.183 ± 0.007	9.153 ± 0.39	1.5 ± 0.1	*P* = 0.001
	B	0.088 ± 0.023	4.380 ± 0.36	1.6 ± 0.1	
	C	0.085 ± 0.006	4.240 ± 0.29	1.6 ± 0.1	

200 (*n* = 3)	A	0.260 ± 0.008	12.980 ± 0.41	1.6 ± 0.0	*P* = 0.003
	B	0.157 ± 0.011	7.873 ± 0.55	1.8 ± 0.1	
	C	0.163 ± 0.031	8.165 ± 0.81	1.4 ± 0.0	

500 (*n* = 3)	A	0.598 ± 0.067	29.900 ± 0.74	1.8 ± 0.1	*P* = 0.000
	B	0.246 ± 0.006	12.293 ± 0.87	1.6 ± 0.0	
	C	0.296 ± 0.020	14.782 ± 0.73	1.7 ± 0.1	

1000 (*n* = 3)	A	1.125 ± 0.089	57.653 ± 5.90	1.7 ± 0.2	*P* = 0.010
	B	0.451 ± 0.031	22.527 ± 2.59	1.8 ± 0.0	
	C	0.761 ± 0.026	38.033 ± 2.62	1.6 ± 0.2	

A: ammonium sulfate DNA extraction method; B: Pinpoint Slide DNA Isolation System; C: TaKaRa DEXPAT.

*Analysis of variance.

^¶^Mean value ± standard deviation.

**Table 2:** Comparison of DNA yield and purity extracted using three different methods from various numbers of PTC cells on archival smear slide.

No. of cells	Extraction method	^¶^DNA concentration (ng/*μ*L)	^¶^Total DNA yield (ng)	^¶^Purity A_260_/A_280_	*P* value*
25 (*n* = 3)	A	0.013 ± 0.002	0.652 ± 0.081	1.6 ± 0.1	* P* = 0.001
	B	0.001 ± 0.001	0.070 ± 0.041	1.0 ± 0.0	
	C	0.004 ± 0.002	0.190 ± 0.122	1.4 ± 0.2	

50 (*n* = 3)	A	0.059 ± 0.007	2.975 ± 0.337	1.7 ± 0.2	*P* = 0.000
	B	0.007 ± 0.003	0.327 ± 0.175	1.2 ± 0.1	
	C	0.017 ± 0.006	0.834 ± 0.277	1.3 ± 0.2	

100 (*n* = 3)	A	0.104 ± 0.008	5.184 ± 0.388	1.5 ± 0.2	*P* = 0.001
	B	0.056 ± 0.007	2.777 ± 0.356	1.6 ± 0.2	
	C	0.085 ± 0.006	4.241 ± 0.291	1.6 ± 0.3	

200 (*n* = 3)	A	0.179 ± 0.008	8.947 ± 0.413	1.6 ± 0.2	*P* = 0.000
	B	0.075 ± 0.011	3.745 ± 0.550	1.8 ± 0.3	
	C	0.136 ± 0.016	6.797 ± 0.812	1.4 ± 0.2	

500 (*n* = 3)	A	0.291 ± 0.015	14.560 ± 0.741	1.8 ± 0.1	*P* = 0.000
	B	0.139 ± 0.017	6.941 ± 0.871	1.6 ± 0.0	
	C	0.190 ± 0.015	9.486 ± 0.732	1.7 ± 0.1	

1000 (*n* = 3)	A	0.406 ± 0.009	20.280 ± 0.457	1.7 ± 0.0	*P* = 0.020
	B	0.242 ± 0.052	12.115 ± 2.585	1.8 ± 0.1	
	C	0.307 ± 0.052	15.347 ± 2.623	1.6 ± 0.1	

A: ammonium sulfate DNA extraction method; B: Pinpoint Slide DNA Isolation System; C: TaKaRa DEXPAT; PTC: papillary thyroid carcinoma.

*Analysis of variance.

^¶^Mean value ± standard deviation.

**Table 3:** Successful **β*-globin* gene amplification frequency using DNA extracted from various numbers of PTC cells on archival smear slides using the three different methods.

No. of cells	A		B		C	
	No. of **β*-globin* amplification	*P* value*	No. of **β*-globin* amplification	*P* value*	No. of **β*-globin* amplification	*P* value*
25 (*n* = 10)	9	0.011	0	0.002	3	0.206
50 (*n* = 10)	9	0.011	3	0.206	4	0.527
100 (*n* = 10)	10	0.002	8	0.058	8	0.058
200 (*n* = 10)	10	0.002	8	0.058	8	0.058
500 (*n* = 10)	10	0.002	10	0.002	9	0.011
1000 (*n* = 10)	10	0.002	10	0.002	10	0.002

A: ammonium sulfate DNA extraction method; B: Pinpoint Slide DNA Isolation System; C: TaKaRa DEXPAT; PTC: papillary thyroid carcinoma. *Two-tailed Fisher's exact test.
